# Influence of Synthesis Temperature on the Growth and Surface Morphology of Co_3_O_4_ Nanocubes for Supercapacitor Applications

**DOI:** 10.3390/nano7110356

**Published:** 2017-10-31

**Authors:** Rashmirekha Samal, Barsha Dash, Chinmaya Kumar Sarangi, Kali Sanjay, Tondepu Subbaiah, Gamini Senanayake, Manickam Minakshi

**Affiliations:** 1 Academy of Scientific and Innovative Research (AcSIR), CSIR-Institute of Minerals and Materials Technology (CSIR-IMMT) Campus, Bhubaneswar 751013, India; samal.rashmirekha1@gmail.com (R.S.); kalisanjay@gmail.com (K.S.); 2 CSIR-Institute of Minerals and Materials Technology, Bhubaneswar 751013, India; sarangi.ck@gmail.com; 3 Office of the R&D, K. L. University, Vaddeswaram 522502, Guntur, India; tsubbaiah@yahoo.com; 4 School of Engineering and Information Technology, Murdoch University, Murdoch, WA 6150, Australia; gamini.senanayake@murdoch.edu.au

**Keywords:** Co_3_O_4_ nanostructures, morphology, temperature, synthesis, capacitors

## Abstract

A facile hydrothermal route to control the crystal growth on the synthesis of Co_3_O_4_ nanostructures with cube-like morphologies has been reported and tested its suitability for supercapacitor applications. The chemical composition and morphologies of the as-prepared Co_3_O_4_ nanoparticles were extensively characterized using X-ray diffraction (XRD) and transmission electron microscopy (TEM). Varying the temperature caused considerable changes in the morphology, the electrochemical performance increased with rising temperature, and the redox reactions become more reversible. The results showed that the Co_3_O_4_ synthesized at a higher temperature (180 °C) demonstrated a high specific capacitance of 833 F/g. This is attributed to the optimal temperature and the controlled growth of nanocubes.

## 1. Introduction

Studies on the effects of morphology and crystal growth of the material synthesized on the micro- and nanoscale have been researched extensively since the discovery of carbon nanotube (CNT) [1]. It is well known that the shape and growth structure of the nanostructured materials strongly influence the properties, which are key factors to their performance in electrical applications [2,3,4,5,6,7,8,9]. Therefore, the application of one-dimensional structures, such as nanowires, nano-belts, and nano-tubes of different nano-materials are increasing due to the demand in technology owing to their excellent characteristics [3,4,5,6,7,8,9]. In the recent years, among the transition metal oxides, cubic Co_3_O_4_, is in much demand because of its broad practical applications in several important technological fields, such as solid-state sensors [10], electro chromic devices [11], heterogeneous catalysts [12], solar energy absorber [13], energy storage devices [14], and pigments [15]. The synthesis of spinel Co_3_O_4_ has been the primary objective for material chemists [16,17]. In these reported articles, a range of synthesis routes to produce spinel Co_3_O_4_ have been proposed, such as namely, the thermal decomposition of a solid cobalt nitrate (380 °C) [18], chemical spray pyrolysis (350–400 °C) [19,20], chemical vapour deposition (CVD, 550 °C) [21], and the traditional sol-gel method (above 260 °C) [22]. It is also reported that porous nanotubes of Co_3_O_4_ can be synthesized by microemulsion method [23], and Co_3_O_4_ nanorods can be achieved by tweaking traditional molten salt synthesis [24] and solvothermal method [25]. Moreover, monodispersed Co_3_O_4_ cubic nanocrystals are reported to be obtained through the salt-solvent process [26]. On the other hand, nanostructured Co_3_O_4_ spherical particles that are formed by the aggregation of much finer particles in homogeneous aqueous solutions had also been reported [27]. Cubic Co_3_O_4_ nanoparticles were reported to be successfully synthesized by a hydrothermal [28] as well as conventional method [29]. The mechanism of the cobalt nanocubes formation is proposed to be “surface wrapping” mechanism. Notably, all of the above said literature are limited to the synthetic technique but the research into the influence of temperature on the growth and surface morphology of Co_3_O_4_crystallites and its redox reactions are not widely researched. Interestingly, the temperature is one of the primary factor controlling the size and shape of the particles that influence the material characteristics for electrochemical applications.

The electrochemical performance of Co_3_O_4_ shown in the literature are varied and influenced by several factors, including particle size, surface morphology, and the ability of particles to adhere to conductive substrates. Recently, Co_3_O_4_ self-supported nano-crystalline arrays that grown directly on conductive substrates exhibited better electrochemical performance [30,31,32]. These studies intrigued us to study the influence of synthesis temperature in a hydrothermal route on the surface morphology of Co_3_O_4_ crystallites and to examine the corresponding redox reactions. Herein, interestingly, we have successfully synthesised Co_3_O_4_ crystallites at different temperatures between 100 °C and 180 °C through hydrothermal route. The structural determination and the morphologies of the Co_3_O_4_ crystallites were analysed through X-ray diffraction and electron microscopies. The significance and novelty of the current work lie in the effect of synthetic temperature on the crystal growth of the cobalt oxide nanostructure. The structural evolution of the crystal at different temperatures gives the purity of the compound with improved energy storage characteristics. The new insights into the synthesis temperature and its influence in redox reactions were observed through cyclic voltammetry experiments. The preliminary studies would bring the importance of temperature and its redox behaviour, which could be useful for supercapacitor applications.

## 2. Materials and Methods

### 2.1. Materials

Cobalt(II) nitrate monohydrate (Co(NO_3_)_2_·H_2_O), urea ((H_2_N)_2_CO), absolute ethanol, acetone, and nafion, were of analytical reagent grade and obtained from SD Fine Chemicals Ltd, Mumbai, India. They were used without further purification. Double-distilled water was used for solution preparation.

### 2.2. Synthesis Procedure

In a typical synthesis procedure, Co(NO_3_)_2_·H_2_O (0.291 g) and urea (0.3 g) was dissolved in 50 mL of deionised water under stirring for 30 min at room temperature. The resultant solution was then transferred to a 60 mL Teflon lined stainless steel autoclave. The synthesis temperature was varied from 100–180 °C for 72 h. The resulted precipitate obtained at different temperature were centrifuged, washed with distilled water and ethanol to remove the ions possibly remaining in the final powders, and finally dried under vacuum at 60 °C overnight.

### 2.3. Characterizations

The physical characterization of the synthesized samples was carried out in different analytical instruments such as X-ray diffraction (XRD), field emission scanning electron microscope (FESEM), Infra-red spectroscopy (FT-IR), and Raman spectroscopy. The XRD patterns were generated in X’Pert PRO PAN analytical diffractometer (Model: PAN ANALYTICAL PW 1830 (Philips, Almelo, The Netherlands) using Cu-Kα radiation in the 2θ range of 10° to 80°. Transmission Electron Microscope (Model: FEI, TECNAI G^2^ 20, TWIN, (FEI (Philips), Alemlo, The Netherlands) coupled with elemental anlysis chamber operated at 200 kV was used to study the internal features of the synthesised samples. For TEM analysis, a very dilute suspension of the synthesised sample was put on carbon coated Cu grids after dispersing in acetone medium. The Field Emission Scanning Electron Microscope (FESEM) measurements were made in Zeiss, SupraTM 55 model scanning electron microscope (Carl Zeiss B. V. Sliedrecht, The Netherlands). To prepare the FESEM samples, purified nano powders were first dispersed in ethanol and then diluted, followed by placing a droplet of the solution onto aluminium foil. The grid was then dried in desiccators for one day before imaging. Infrared spectra of the samples formed in KBr platelets were recorded with JASCO FT-IR 420 spectrometer (JASCO, Tokyo, Japan). The Raman spectra were recorded using RENISHAW Raman Spectrometer (RENISHAW, London, UK) where an argon ion laser beam was used as the excitation source at 540 nm.

Electrochemical measurements such as cyclic voltammetry (CV) was performed using a CHI 680C workstation (CH Instrument, Austin, TX, USA) with a typical three-electrode cell equipped with an N_2_ gas flow system. The Co_3_O_4_ samples synthesized at different temperatures were deposited on a glassy carbon surface served as a working electrode, an Ag/AgCl electrode served as a reference electrode and a platinum wire as a counter electrode. The electrolyte was 1 M KOH aqueous solution. The active mass of the working cobalt oxide electrode was approximately 5 mg.

## 3. Results and Discussion

### 3.1. Physical Characterization of Co_3_O_4_ Crystallites

#### 3.1.1. XRD Analysis

**Figure 1:**
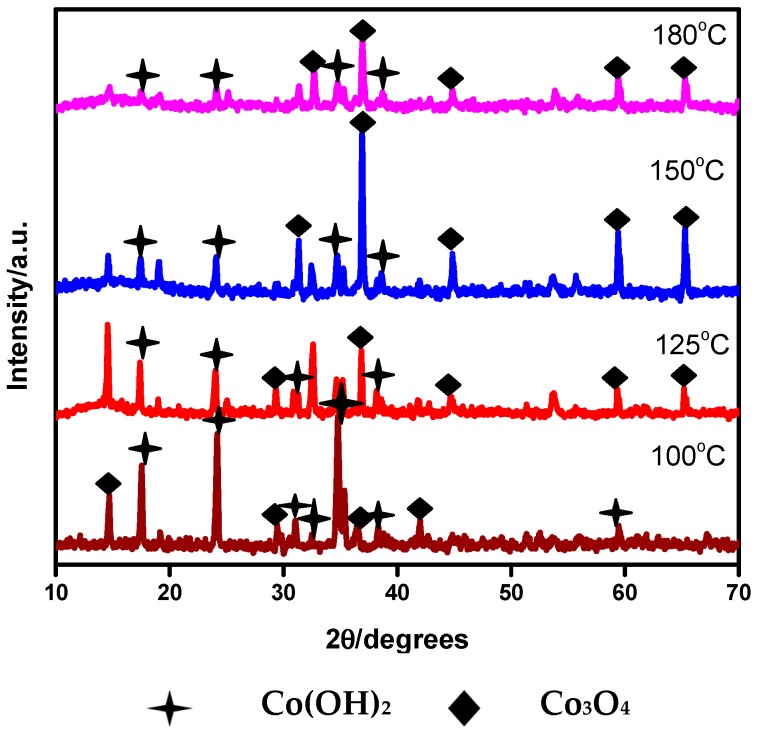
X-ray diffraction (XRD) patterns of the Co_3_O_4_ synthesized at a different temperature.

The XRD patterns of the Co_3_O_4_ samples synthesized at various temperatures are shown in Figure 1. All of the X-ray reflections in Figure 1 can be indexed to mixed phases of hexagonal β-Co(OH)_2_ (Wyckoff positions of space group: P-3m1 (164); 1a and cubic β-Co_3_O_4_ (Wyckoff positions of space group: Fd-3m (227); 16c, which are in good agreement with the available database (JCPDS No. 01-074-1057) and (JCPDS No. 03-065-3103).

No diffraction peaks corresponding to α-phase compounds are detected in the XRD pattern. This indicates that the obtained product is of high pure β phase material that could be achieved at low synthesis temperatures. The absence of X-ray diffraction peaks corresponding to other second phases, such as CoO and CoOOH, confirms that the final product is a mixture of β-Co_3_O_4_ and β-Co(OH)_2_. The sample synthesized at a relatively higher temperature exhibits peaks corresponding to β-Co_3_O_4_ at a higher intensity (in Figure 1), while the cobalt hydroxide phase peaks are converted to cobalt oxide phase. This shows that the optimal temperature for the synthesis of β-Co_3_O_4_ is around 180 °C. To gain more insight into the crystal structure, a range of spectroscopy and microscopic analyses have been performed and discussed in the subsequent sub-sections.

#### 3.1.2. Infra-Red and Raman Analysis

**Figure 2:**
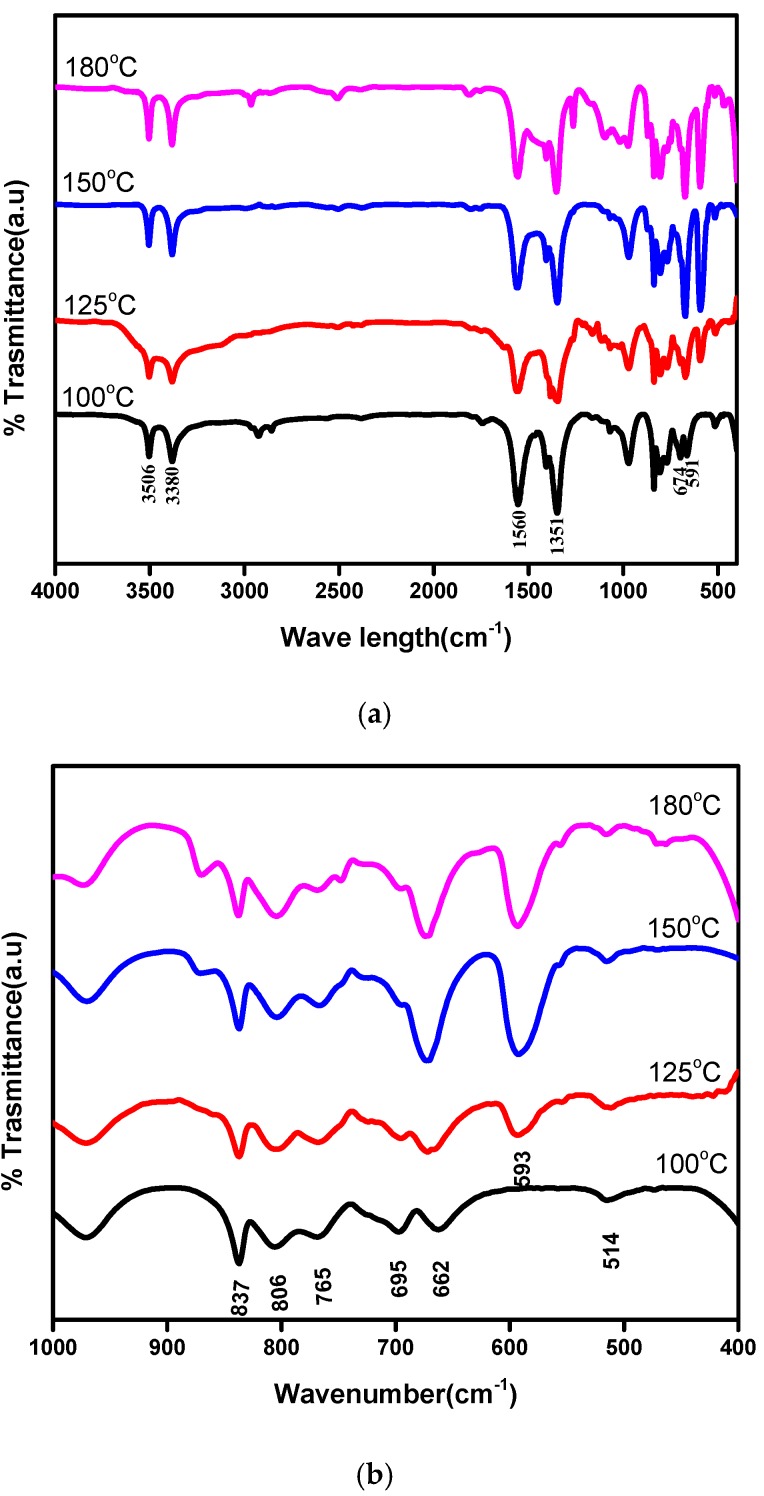
Infra-red spectra of the Co_3_O_4_ synthesized at different temperatures showing (**a**) near (1000–4000 cm^−1^) and mid IR (400–1000 cm^−1^) and (**b**) mid IR region ((400–1000 cm^−1^).

The IR spectrum of the as-prepared Co_3_O_4_nanocrystals at different temperatures is shown in Figure 2. To get more insights in the mid IR regions, magnified spectra is also presented in Figure 2b. The absorption peaks at 674 and 591 cm^−1^ are assigned to the ν (Co–O) modes [33], which indicates the formation of cobalt oxide nanocrystals. It is observed that these two peaks started to evolve with an increase in synthesis temperature, indicating the formation of cobalt oxide is becoming significant at higher temperatures. The broad band centred at 3566 cm^−1^, 3380 cm^−1^ and the peak at 1632 cm^−1^ correspond to the stretching and bending modes of the hydroxyls, respectively [34].

The peaks at about 1560 and 1051 cm^−1^ are attributed to the carbonate groups originating from the reaction of oxide with air forming CO_2_ during the analysis procedure. The peaks at 1351 and 865 cm^−1^ are related to the ν3 and ν2 vibrational modes of NO^3−^ intercalated in the interlayers [35]. The band at 514 cm^−1^ corresponds totheγ-Co–O–H bond vibration in the cobalt hydroxide.

**Figure 3:**
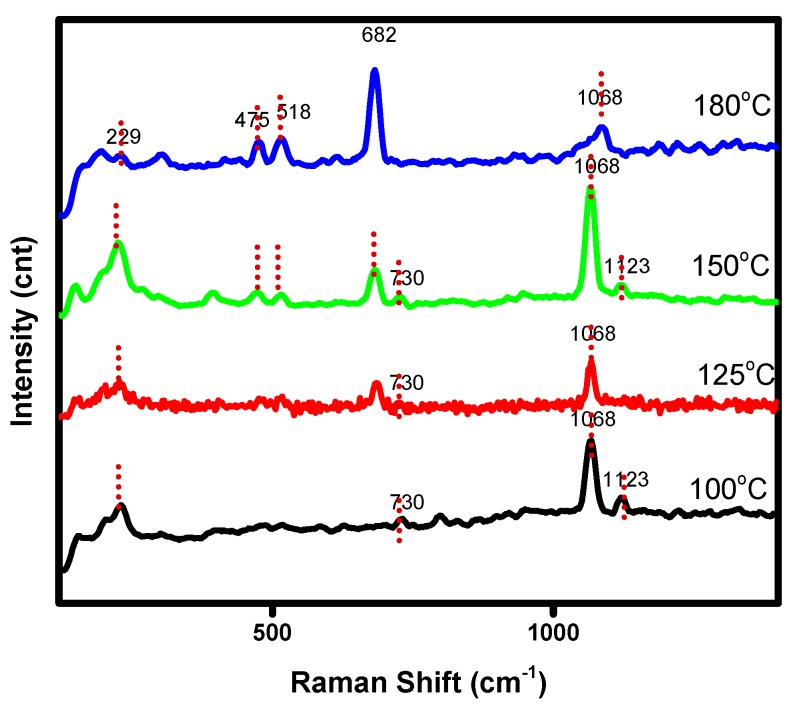
Raman spectra of cobalt oxide obtained at a different temperature.

To get further insights on the bonding structure Raman microscopy was carried out on these samples. The characteristic Raman bands for Co_3_O_4_ positioned at 475 cm^−1^, 518 cm^−1^, and 682 cm^−1^ are shown in Figure 3. These bands appear to be significant for the sample synthesized at 180 °C. The intensity of the characteristic bands progressively increased from lower to higher synthetic temperature. Simultaneously, the bands at 229 cm^−1^ and 1068 cm^−1^ are the characteristic bands of cobalt hydroxide Co(OH)_2_. These bands are prominent in the sample synthesized at 100 °C where the major phase is the cobalt hydroxide. The intensity of these bands corresponding to Co(OH)_2_ decreases slowly when the synthesis temperature increased from 100 °C to 180 °C. From the Raman analysis, it is clear that the cobalt oxide phase gradually becomes prominent when the synthesis temperature increases on the other hand cobalt hydroxide phase diminish. This confirms the optimal temperature is 180 °C for the Co_3_O_4_ product to be produced.

#### 3.1.3. Field Emission Scanning Electron Microscopy Analysis (FESEM)

**Figure 4:**
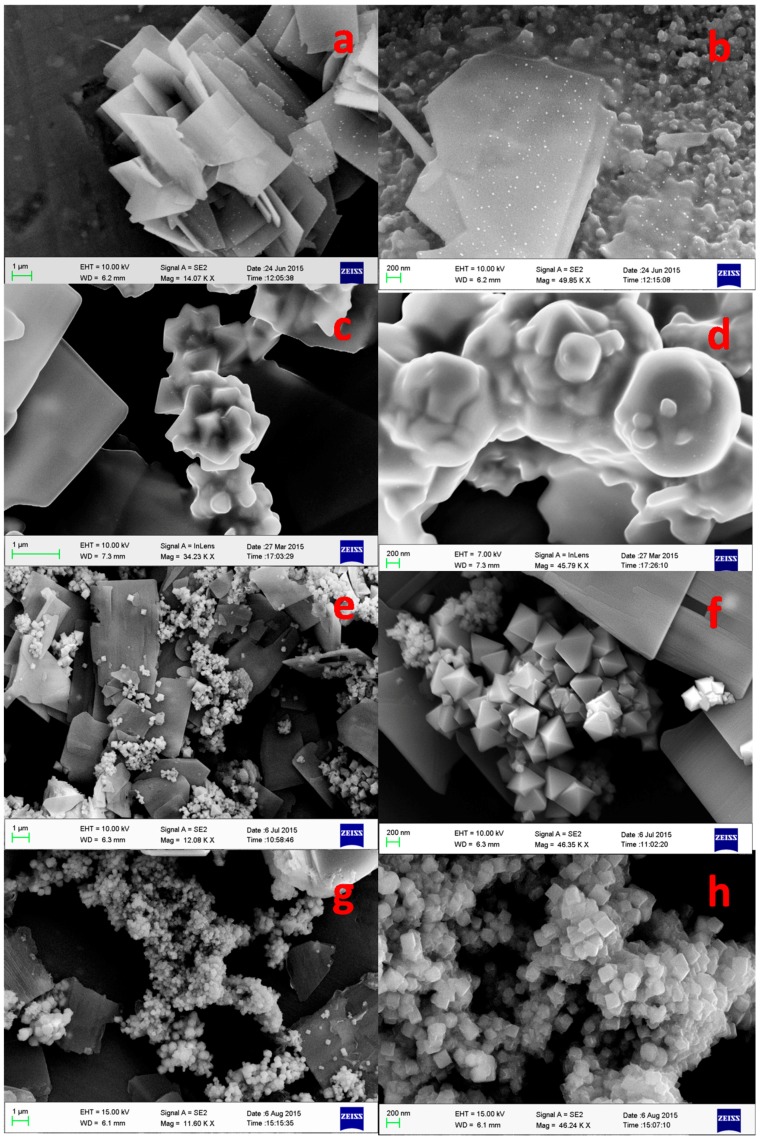
FE-SEM images of cobalt oxides at (**a**,**b**) 100 °C, (**c**,**d**) 125 °C, (**e**,**f**) 150 °C, and (**g**,**h**) 180 °C at low (**left**) and high (**right**) magnifications.

The morphology of the as-synthesized products at different temperatures is shown in Figure 4. The FE-SEM images (Figure 4a,b) show that the prepared oxide has layered morphology and consists of a large quantity of flake-like nanostructures having the initial growth of cubes on its surface. The particles are shown to have a uniform size, lower specific area that is associated with a suitable diameter and thickness. The nano flakes have a thickness of less than about 10–20 nm and a size of 2–3 µm in the other two dimensions. While increasing the synthesis temperature from 100 °C to 125 °C, interestingly, the surface morphology changes gradually from flake to nodular growth (Figure 4c,d). The particles are agglomerated that may not be suitable for any diffusion of ions, which is a pre-requisite for electrochemical applications. Whereas, when the synthesis temperature is increased to 150 °C (Figure 4e,f) the nanostructured material appears to be in a pyramidal-like shape but it appears to be in less uniformity. Upon, further increasing the temperature from 150 °C to 180 °C (Figure 4g,h) a large quantity of cube-like nanostructures are found to be deposited on the surface of the material. The formation of cubes could be due to the change in the pH of the solution in presence of urea along with the change in reaction temperature [36].

The possible second phase reactions involved in the synthesis of Co_3_O_4_ nanostructures at lower temperatures between 100 °C and 150 °C can be summarized as:

Co(NO_3_)_2_ → Co^2+^ + NO_3_^2−^ (1)

(H_2_N)_2_CO + H_2_O → 2NH_3_ + CO_2_ (2)

NH_3_ + H_2_O → NH^4+^ +OH^−^ (3)

Co^2+^+ 2OH^−^ → Co(OH)_2_ (4)

It is clear from Figure 4 that the morphology of the obtained product has been progressively changed during the thermal treatment in presence of urea. The obtained Co_3_O_4_ crystalline size and its growth inferred from the microscopic images are found to be in good conformity with the XRD results.

#### 3.1.4. TEM Analysis

**Figure 5:**
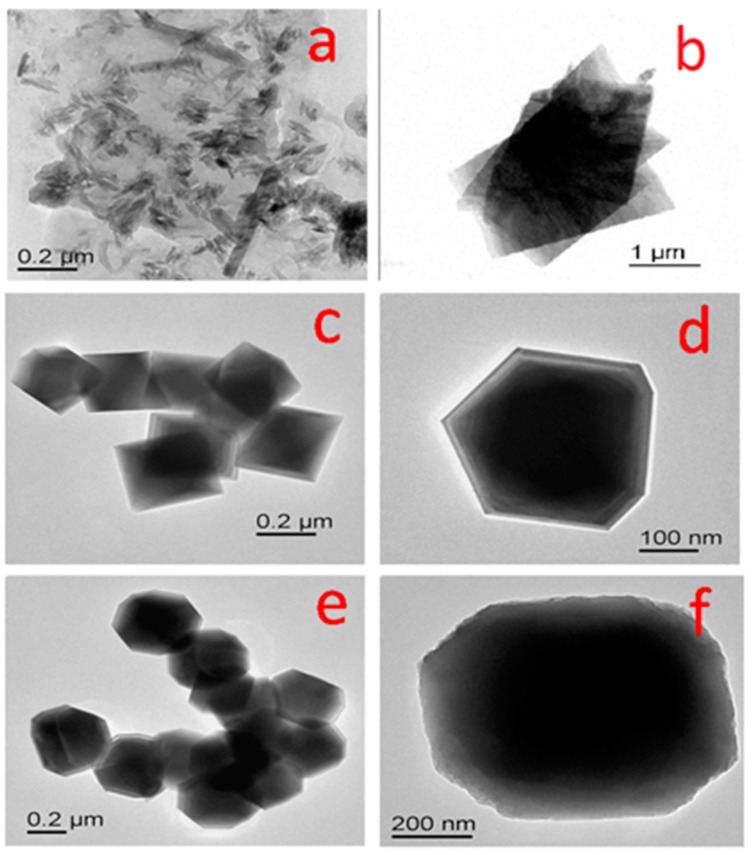

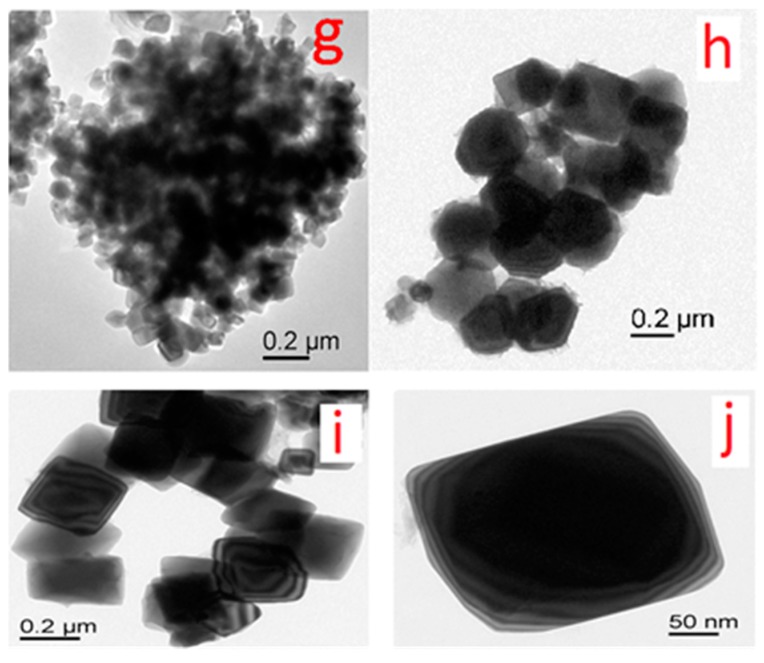
TEM images of cobalt oxide nano particles synthesized at different temperatures (**a**,**b**) 100 °C; (**c**,**d**) 125 °C showing pyramidal shape; (**e**,**f**) 150 °C showing transitional shape; and (**g**–**j**) 180 °C showing cubical geometry.

**Figure 6:**
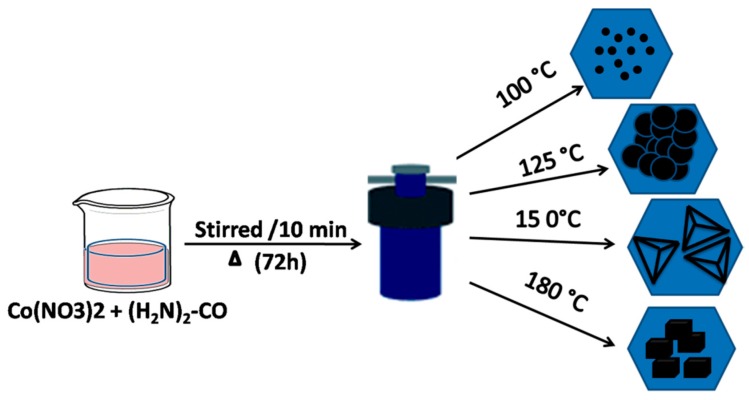
Schematic representation of the process and morphology of cobalt oxide at different temperature.

The morphology of the as-synthesized product and its crystal growth upon synthesis temperature was further characterized by the transmission electron microscopy (TEM) technique. Figure 5 shows the representative TEM images of the Co_3_O_4_ powders that were synthesized at different temperatures. The samples prepared at 100 °C exhibited nano flake like structure as seen in Figure 5a. A gradual structural evolution from hydroxide to the cobalt oxide nanoparticles has been found to form at 180 °C (Figure 5g–j). The TEM images shows the evolution of fine Co_3_O_4_ nanocubes, initially on the surface of cobalt hydroxide (Figure 5a,b), while taking several other shapes ,including pyramidal (Figure 5c,d), and transitional (Figure 5e,f), before forming cubical like structure (Figure 5g–j). To summarise this, the structural evolution of the cobalt oxide on the surface of cobalt hydroxide β-Co(OH)_2_ [37] can be explained pictorially in a schematic diagram presented in Figure 6.

### 3.2. Electrochemical Characterization

#### Cyclic Voltammetry (CV) of Cobalt Oxide as Working Electrode for Supercapacitor Applications

The electrolytes used in the electrochemical analysis such as cyclic voltammetry must possess an electrochemical stability with a safe voltage window before any decomposition occurs. The resistance of the cobalt oxide-working electrode depends on the resistivity of the electrolyte used and the size of the ions from the electrolyte that diffuse into and out of the pores of the electrode particles. For instance, in the case of, organic electrolytes have a higher resistance, and hence there will be a power drop but that can be usually offset by the gain in higher cell voltage. The organic electrolytes also have safety concerns, as they are flammable. Whereas, this is not the case for an aqueous electrolyte, such as sodium hydroxide, potassium hydroxide, or sulphuric acid [38,39]. Moreover, aqueous electrolytes are cheaper, easier to purify and have a lower resistance, but they limit the cell voltage to typically 1V, thereby limiting the maximum achievable power [40]. Therefore, with these issues in mind, in the current work, the concentration of 1.0 M aqueous KOH electrolyte was prepared in freshly prepared double distilled water and used as an electrolyte for examining cobalt oxide as a potential electrode for supercapacitor applications.

**Figure 7:**
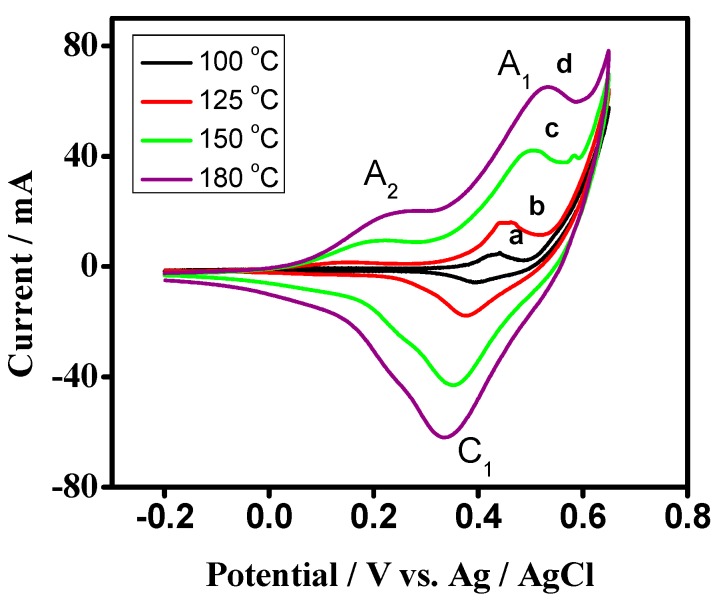
Cyclic voltammetric (CV) curves of cobalt oxide electrode synthesized at different temperatures (**a**) 100 °C, (**b**) 125 °C, (**c**) 150 °C, and (**d**) 180 °C at a scan rate of 50 mV s^−1^. CV peaks showed typical redox behaviour and becoming more prominent at a higher temperature.

**Figure 8:**
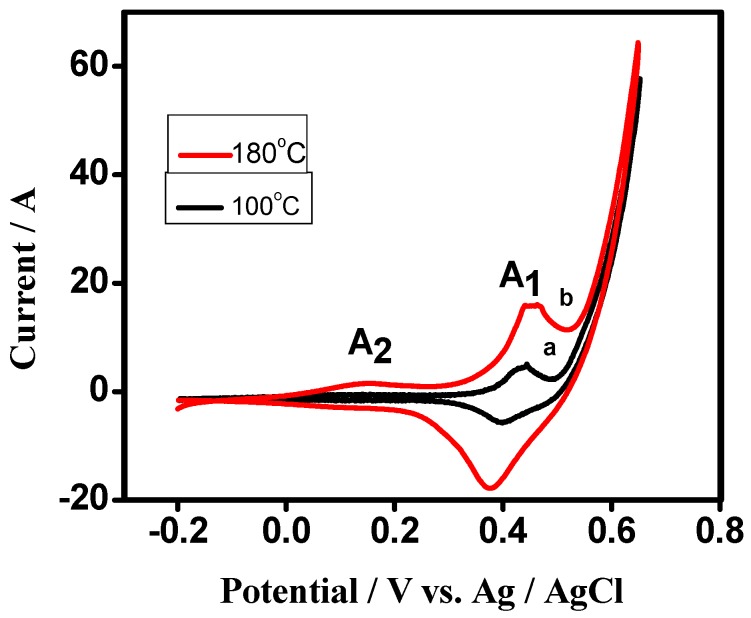
Cyclic voltammetric (CV) curves of cobalt oxide electrode synthesized at (**a**) 100 °C, (**b**) 125 °C, at a scan rate of 50 mV s^−1^ (magnified curves of Figure 7a,b).

The influence of temperature in the synthesis of Co_3_O_4_ crystallites and testing its suitability for energy storage applications was examined by keeping the scan rate and potential window constant. Figure 7 exhibits the typical CV curves of cobalt oxide (Co_3_O_4_) electrode at scan rate 50 mV s^−1^ within the potential range of +800 to −200 mV vs. Ag/AgCl in KOH electrolyte for the samples that were synthesized at a different temperature. All of the tested samples exhibit two oxidation curves (A_1_ and A_2_) in the anodic scan but one reduction curve (C_1_), while reversing the scan to cathodic direction. During the cathodic scan, Co_3_O_4_ is reduced to form CoOOH. In the anodic scan, CoOOH undergoes a change in the oxidation state of the Co atom forming CoO_2_ (A_2_) and Co_3_O_4_ (A_1_) governed by faradaic reactions. The voltammogram obtained for the samples synthesized at lower temperature appeared to have redox peaks with a lower intensity and did not show well-resolved redox peaks. Hence, the magnified curves for the samples synthesized at lower temperatures are also shown in Figure 8. When the temperature was increased from 100 °C to 180 °C, the redox peaks were seen noticeably, in Figure 7, and have a well defined shape, illustrating that the material is more reversible and the reactions are significant when the material is dominated by Co_3_O_4_ nanoparticles. The specific capacitance ‘*C*’ was calculated [41] from the relation:

(5) $$Cs=\frac{\int_{V1}^{V2}iV\;dv\;}{2\left(V2-V1\right)\;\nu \;m}$$

where *C*s is the specific capacitance in F/g. *V*1 and *V*2 are the cut off potential, and *iV* is the instantaneous current.$\;\int_{V1}^{V2}iV\;dv\;$ is the integrated area of the CV curve. (*V*2 − *V*1) is the potential window in *V*. *m* is the mass of the active material in the single electrode in g, which is the mass difference of the working electrode. *ν* is the potential scan rate (v/s) and the factor 2 corrects the fact that the above integration area includes both the positive scan and negative scan. The maximum specific capacitance was obtained for the sample synthesized at 180 °C.

The predicted values of standard reduction potentials of the proposed redox couples [41] given in Equations (6) and (7) are 0.437 and 0.428 V, respectively, at 100 °C. These values shifted to 0.442 and 0.346 V at 180 °C respectively, and the following redox reactions are considered to be governing the pseudocapacitive properties of Co_3_O_4_.

Co_3_O_4_ + H_2_O + OH^−^ ↔ 3CoOOH + e^−^   (peaks A_1_/C_1_) (6)

CoOOH + OH^−^ ↔ CoO_2_ + H_2_O + e^−^    (peak A_2_) (7)

The electrochemical behaviour of Co_3_O_4_ in Figure 7 and Figure 8 exhibits the quasi-reversible faradaic process that involves electron transfer process in Co^2+^/^3+^ redox couple involving the ability of OH^−^ to be reversibly intercalated into the reduced form of Co_3_O_4_ for the improved charge storage (forming COOH; peak A_1_/C_1_), which is attributed to pseudocapacitance. However, the peak A2 (Equation (7)) is raised from the formation of cobalt oxide (CoO_2_) and adsorption of ions on the near-surface corresponding to the non-faradaic process thatis not reversible during the reduction process but contributes to the capacitance. Therefore, the above studies indicate that reduction of Co_3_O_4_ is reversible while the further reduction of CoOOH forms a mixture of CoO_2_ and adsorption of OH^−^ ions, which is irreversible. Based on this mechanism, the value of specific capacitances obtained for the samples synthesized at 100 °C, 125 °C, 150 °C, and 180 °C is 45 F/g, 155 F/g, 506 F/g, 833 F/g, respectively, at a san rate 50 mV s^−1^. Overall, the enhanced performance of the Co_3_O_4_ is influenced by the synthesis temperature, pH of the solution and the role of urea as a fuel in the typical hydrothermal process [42].

## 4. Conclusions

A hydrothermal route for the synthesis of Co_3_O_4_ nanocubes has been reported for supercapacitor applications. The effect of synthesis temperature on the crystal growth of Co_3_O_4_ was studied systematically and the preliminary studies of its electrochemical activity showed they are an excellent material for energy storage. The Co_3_O_4_ nanostructures exhibit cube-like morphology and have comprised of nanocubes with the increase in synthesis temperature and in the presence of urea as fuel. The studies showed that the higher the synthesis temperature, the faster the growth rate, and hence the obtained nanocubes. TEM analysis confirmed the morphology that the individual Co_3_O_4_ nanocubes consist of aggregated nanocrystals. Co_3_O_4_ nanocubes demonstrated a high capacitance value of 833 Fg^−1^ and the materials synthesized at an optimum temperature of 180 °C showed a high reversibility with adsorption and desorption of ions from the aqueous electrolyte.
